# A Rapid-Response Humoral Vaccine Platform Exploiting Pre-Existing Non-Cognate Populations of Anti-Vaccine or Anti-Viral CD4+ T Helper Cells to Confirm B Cell Activation

**DOI:** 10.1371/journal.pone.0166383

**Published:** 2016-11-18

**Authors:** Thomas Hills, Phillip G. Jakeman, Robert C. Carlisle, Paul Klenerman, Leonard W. Seymour, Ryan Cawood

**Affiliations:** 1 Department of Oncology, University of Oxford, Oxford, United Kingdom; 2 Department of Biomedical Engineering, University of Oxford, Oxford, United Kingdom; 3 Peter Medawar Building for Pathogen Research, University of Oxford, Oxford, United Kingdom; Midwestern University, UNITED STATES

## Abstract

The need for CD4+ T cell responses to arise *de novo* following vaccination can limit the speed of B cell responses. Populations of pre-existing vaccine-induced or anti-viral CD4+ T cells recognising distinct antigens could be exploited to overcome this limitation. We hypothesise that liposomal vaccine particles encapsulating epitopes that are recognised, after processing and B cell MHCII presentation, by pre-existing CD4+ T cells will exploit this pre-existing T cell help and result in improved antibody responses to distinct target antigens displayed on the particle surface. Liposomal vaccine particles were engineered to display the malaria circumsporozoite (CSP) antigen on their surface, with helper CD4+ epitopes from distinct vaccine or viral antigens contained within the particle core, ensuring the B cell response is raised but focused against CSP. *In vivo* vaccination studies were then conducted in C57Bl/6 mice as models of either vaccine-induced pre-existing CD4+ T cell immunity (using ovalbumin—OVA) or virus-induced pre-existing CD4+ T cell immunity (murine cytomegalovirus—MCMV). Following the establishment of pre-existing by vaccination (OVA in the adjuvant TiterMax^®^ Gold) or infection with MCMV, mice were administered CSP-coated liposomal vaccines containing the relevant OVA or MCMV core CD4+ T cell epitopes. In mice with pre-existing anti-OVA CD4+ T cell immunity, these vaccine particles elicited rapid, high-titre, isotype-switched CSP-specific antibody responses—consistent with the involvement of anti-OVA T helper cells in confirming activation of anti-CSP B cells. Responses were further improved by entrapping TLR9 agonists, combining humoral vaccination signals ‘one’, ‘two’ and ‘three’ within one particle. Herpes viruses can establish chronic infection and elicit significant, persistent cellular immune responses. We then demonstrate that this principle can be extended to re-purpose pre-existing anti-MCMV immunity to enhance anti-CSP vaccine responses—the first description of a strategy to specifically exploit anti-cytomegalovirus immunity to augment vaccination against a target antigen.

## Introduction

Vaccines are arguably our most effective medical technology, combining scientific innovation with a worldwide medical need. Although successful, many of the advances have been pragmatic, such as pathogen attenuation, rather than designed to exploit specific molecular mechanisms [1]. However, effective vaccines for diseases such as malaria and human immunodeficiency virus have eluded existing strategies and promising approaches in these fields are increasingly based on specific mechanistic hypotheses [2].

Here we describe a new humoral vaccine platform designed to allow rapid and efficient activation of antigen-specific B cells. Normally, following antigen internalisation into B cells bearing appropriate B cell receptors (BCR), antigen-derived epitopes are presented on MHCII molecules and costimulatory signals from CD4+ T cells drive B cell activation and maturation [3] [4] [5]. Helper CD4+ T cells can recognise epitopes from the same antigenic protein (cognate) or another protein simultaneously taken up by the B cell (non-cognate). Accordingly, by associating strong MHCII helper epitopes as part of the same vaccine particle as the target antigen, it is possible to confirm antigen-specific B cell activation by pre-existing non-cognate CD4+ T cells that recognise an epitope from a different immunological target. Conjugate vaccines—where carbohydrate B cell antigens are coupled to strong protein carriers—are the archetypal example of a vaccine strategy exploiting this mechanism to enhance antibody responses to weak antigens. However, simply conjugating helper peptides or proteins to target antigens does not reliably enhance anti-target antibody responses. This approach can result in the immune response to the protein carrier dominating over that generated against the target antigen [6] [7] [8].

**Fig 1:**
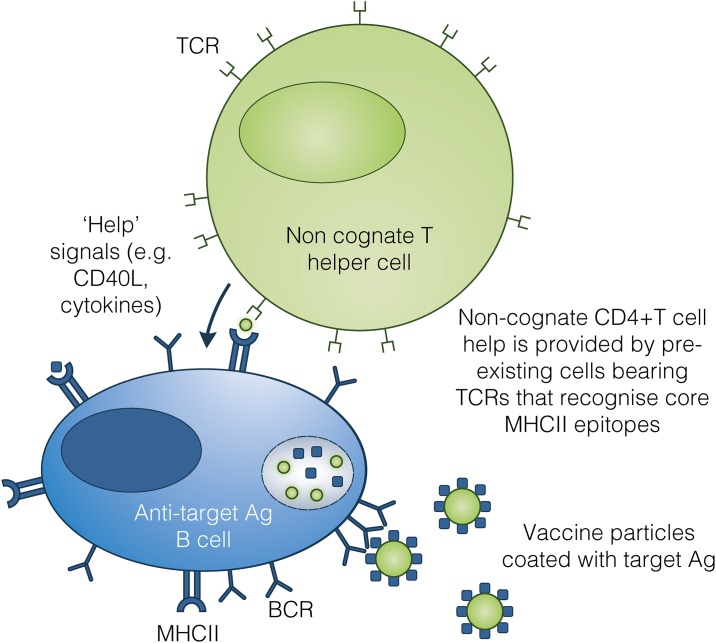
Vaccine mechanism of action. Pre-existing non-cognate CD4+ T cell help is exploited to enhance antibody responses to target antigens. B-cells with BCRs specific for the antigen on the particle surface are engaged by vaccine particles. Following internalisation, endosomal proteases generate peptide fragments from both the surface antigen and the helper antigen entrapped within the vaccine particle. These may be presented on MHC class II to cognate T cells (i.e. those that recognise the same antigen as the BCR—which may be rare for weak antigens) and non-cognate T cells (i.e. those that recognise the strong helper MHCII epitopes). Pre-existing CD4+ T cells that recognise the helper antigen (generated by pre-vaccination or infection) can provide costimulatory signals to the B-cell, resulting in confirmation of antibody production. These signals drive B-cell proliferation, differentiation, antibody production, somatic hypermutation, and isotype switching. Because the specificity of this antibody response was determined at the point of BCR-mediated antigen recognition, the antibody produced will be directed against the target antigen on the vaccine particle surface, and not against the core helper components.

In our strategy, liposomal vaccine particles are engineered to only display target antigens for antibody responses on the liposome surface, while helper CD4+ T cell epitopes from unrelated antigens are entrapped within the liposome. Vaccine particles are recognised and internalised by B cells bearing target antigen-specific BCR, allowing intracellular processing of helper epitopes—previously shielded from the immune system—for presentation on MHCII to non-cognate helper CD4+ T cells (Fig 1). This approach differs from conjugate vaccines in that it seeks to focus the B cell response on the surface-bound target antigen, minimising the antibody response to the core helper epitopes.

We hypothesise that pre-existing helper CD4+ T cells that recognise helper epitopes entrapped within the liposome particle can be exploited to increase the speed, magnitude, isotype switching, and avidity of antibodies produced against the target antigen. These non-cognate CD4+ T cells could be elicited by previous vaccination or simply by natural exposure to pathogens in the environment. For example, the majority of the human population are chronically infected with HCMV, with seroprevalence reaching as high as 90.8% in those over 80 years old [9]. 1–2% of the CD4+ T cell pool is directed against HCMV epitopes in healthy seropositive individuals, but this can reach more than 40% [10] [11] [12]. Individual clones can represent 0.3–1.5% of the total peripheral CD4+ T cell population [13]. It would therefore be an appealing strategy to repurpose these cells to support humoral vaccination against unrelated target antigens that we describe here.

Vaccinologists often discuss ‘signals one, two, and three’ as important components of an effective humoral vaccine approach, where signal one and signal two represent antigen-specific BCR-recognition and CD4+ T cell help, respectively. Signal three represents adjuvant stimulation of the antigen-presenting cell (in this case the B cell), normally via Toll-like receptors (TLRs). Accordingly, we encapsulated TLR agonists alongside helper epitopes to generate ‘self-adjuvanting’ vaccine particles capable of co-ordinated delivery of signals one, two, and three to target B cells to determine if that would potentiate activity still further.

## Results

### Pre-existing non-cognate CD4+ T cells can be exploited to enhance antibody responses to target antigens displayed on the surface of vaccine particles

**Fig 2:**
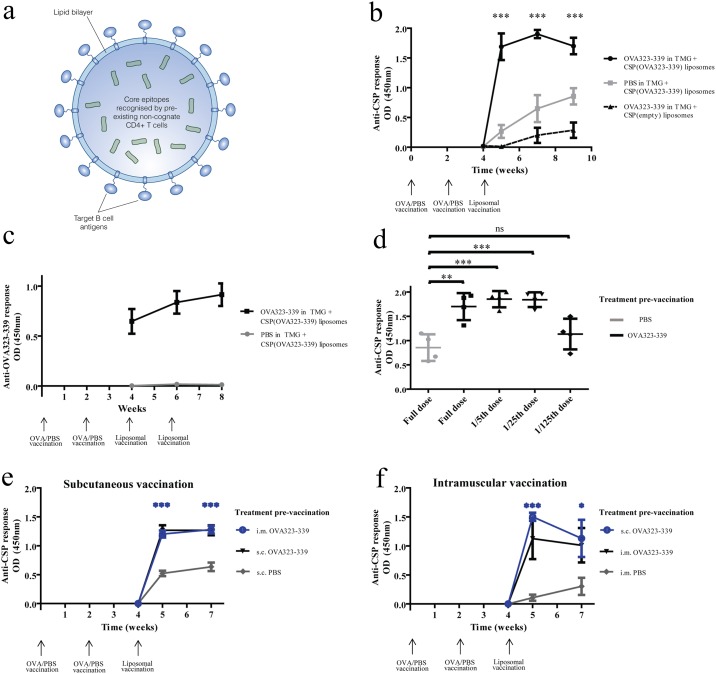
Pre-existing CD4+ T cell immunity to epitopes contained within the core of liposomal vaccine particles enhances the antibody response to non-cognate target antigens on the particle surface. Mice (n = 4) were pre-vaccinated with either PBS or OVA_323-339_ in TMG at week 0 and week 2, prior to vaccination with CSP(OVA_323-339_) liposomes at week 4. The effect of this pre-existing immunity to OVA_323-339_ on anti-CSP antibody responses was measured over weeks 4 to 8 and compared using two-way ANOVA with Bonferroni’s post-test. Asterisks indicate the statistical significance of the difference between OVA_323-339_ + TMG (black circles, solid black line) and PBS + TMG (grey squares, solid grey line) pre-vaccinated groups. This difference was found reproducibly in multiple experiments and a typical result is shown (b). Anti-OVA_323-339_ responses were also measured in both PBS and OVA_323-339_ pre-vaccinated mice administered CSP(OVA_323-339_) liposomes (c). The effect of liposomal particle dose was examined by vaccinating mice (n = 4) with five-fold dilutions of CSP(OVA_323-339_) vaccine (the highest concentration containing 50 μg of CSP and 10 μg of OVA_323-339_). Anti-CSP responses were compared using a one-way ANOVA with Tukey’s post-test for multiple comparisons (d). Pre-vaccination with PBS or OVA_323-339_ in TMG was then performed either intramuscularly or subcutaneously. Subsequently, CSP(OVA_323-339_) liposomal vaccines were then administered either subcutaneously (e) or intramuscularly (f) and mean anti-CSP responses were compared over time using two-way ANOVA with Bonferroni’s post test. Asterisks indicate the difference between groups shown in blue and grey groups at the indicated time points.

To determine whether pre-existing non-cognate T cells could be harnessed to support immune responses to new target antigens on the surface of vaccine particles, liposomal vaccine particles were produced with spatially segregated B and CD4+ T cell antigens. Immunodominant CD4+ T cell epitopes of the model antigen ovalbumin (OVA_323-339_) were incorporated into the liposome core, while the B cell epitope from *Plasmodium falciparum* malaria circumsporozoite protein—five repeats of NANP terminated with a cysteine residue (CSP)–was conjugated to the particle surface (Fig 2a). The resulting liposomal particles were designated CSP(OVA_323-339_) to denote that CSP is on the surface and OVA peptides are entrapped within. Particle size and polydispersity were characterised by dynamic light scattering (S2 Fig), before being used to vaccinate mice without pre-existing immunity to OVA_323-339_ and mice vaccinated with OVA_323-339_ emulsified in TiterMax^®^ Gold (TMG) adjuvant (S1 Fig).

In keeping with the proposed mechanism of action, vaccination with CSP(OVA_323-339_) liposomes in mice pre-vaccinated with OVA_323-339_ elicited anti-CSP responses more quickly, peaking after just a few days, and reaching two-fold higher levels in that time frame when compared to mice pre-treated with PBS (Fig 2b). This enhancement required the presence of OVA_323-339_ in the particle vaccine, as a CSP(empty) liposome control produced substantially lower responses (Fig 2b). Crucially anti- OVA_323-339_ antibody responses were not increased following CSP(OVA_323-339_) vaccination (Fig 2c—grey line), commensurate with our hypothesis that entrapping the OVA_323-339_ helper antigens within the liposome would render them unavailable for BCR binding, and focus the humoral immune response on the target antigen CSP. To define the magnitude of the effect that pre-existing anti-OVA CD4+ T cell immunity could have on B cell responses to CSP(OVA_323-339_) vaccine particles, we assessed the anti-CSP responses to different doses of vaccine particles. The anti-CSP-antibody response produced in mice pre-vaccinated with OVA_323-339_ was not diminished using particle vaccine doses five, or twenty five, -fold lower. In OVA_323-339_ pre-vaccinated mice, a twenty five-fold lower dose of vaccine produced a greater response than a full dose in mice that did not have pre-existing anti- OVA_323-339_ immunity (Fig 2d).

Furthermore, in groups of mice where pre-existing immunity to OVA_323-339_ was generated at sites anatomically distinct to the route of CSP(OVA_323-339_) liposomal vaccine administration, enhanced anti-CSP responses were still seen (Fig 2e and 2f). This suggests that higher responses in OVA_323-339_ pre-vaccinated mice do not arise due to a local immune phenomenon and demonstrates that pre-existing, non-cognate *systemic* immunity can be exploited to enhance antibody responses.

These data show that pre-existing non-cognate CD4+ T cell help generated by OVA_323-339_ vaccination can support rapid, higher titre anti-CSP antibody responses to CSP(OVA_323-339_) liposomes, supporting our hypothesis that repurposing pre-existing CD4+ T cells can potentiate humoral vaccine responses.

### Pre-existing non-cognate CD4+ T cells drive the rapid production of isotype-switched higher affinity antibodies

CD4+ T cell help can increase the magnitude of a developing humoral immune response, but also affects the isotype and avidity of the antibody produced [3]. We hypothesised that mice with pre-existing anti-OVA_323-339_ CD4+ T cells would mount isotype-switched, higher avidity, anti-CSP antibody responses to our vaccine more quickly than PBS pre-vaccinated animals and that the T_H_1/T_H_2 polarisation of the anti-OVA_323-339_ CD4+ T cell population might well regulate the isotype profile of the anti-CSP response.

**Fig 3:**
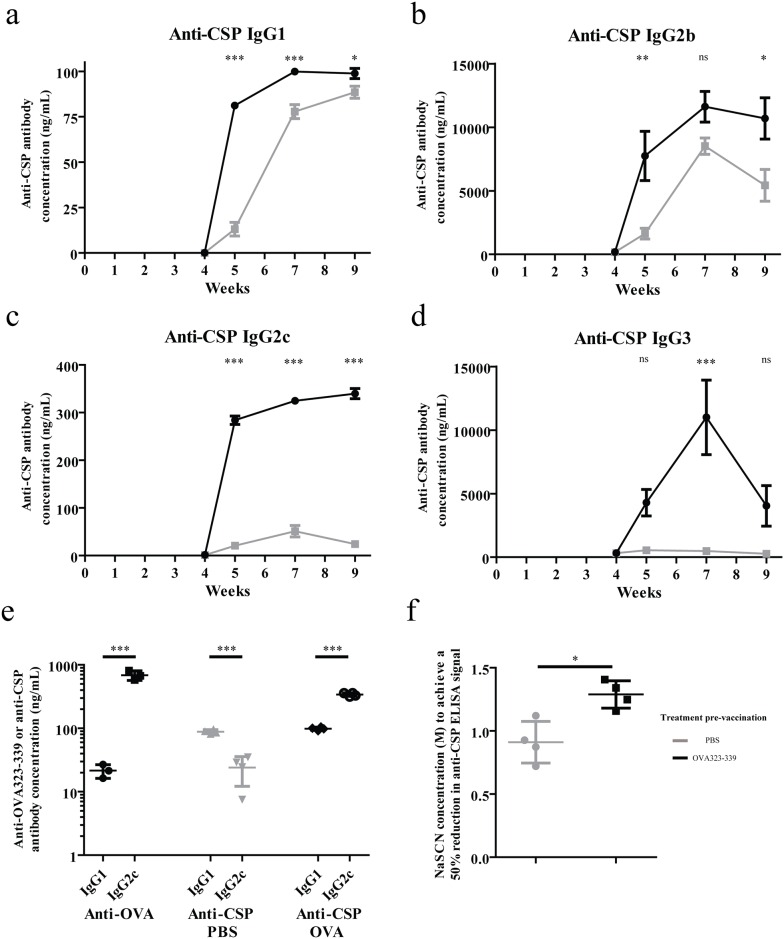
Pre-existing CD4+ T cell immunity to non-cognate core epitopes drives the rapid production of isotype-switched, higher avidity antibodies against target antigens. CSP-specific IgG1 (a), IgG2b (b), IgG2c (c) and IgG3 (d) responses to vaccination with CSP(OVA_323-339_) were quantified in mice (n = 4) pre-vaccinated with either PBS + TMG or OVA_323-339_ + TMG using two-way ANOVA with Bonferroni’s post-test. (e) The anti-OVA_323-339_ IgG1/IgG2c response pattern at the time of liposomal vaccination was compared with the anti-CSP IgG1/IgG2c response four weeks after CSP(OVA_323-339_) vaccination in PBS + TMG or OVA_323-339_ + TMG pre-vaccinated mice (n = 4) using unpaired, two-tailed t tests. If Welch’s correction is used for unequal variances, the significance of difference between the anti-OVA_323-339_ IgG1 and IgG2c responses reduces to * (p < 0.05). (f) The concentration of the chaotropic agent sodium thiocyanate required to decrease the anti-CSP ELISA signal by 50% was determined in serum from mice (n = 4) pre-vaccinated with either PBS or OVA_323-339_ in TMG four weeks after vaccination with CSP(OVA_323-339_) liposomes and compared with unpaired, two-tailed t tests.

As predicted, pre-vaccination with OVA_323-339_ resulted in earlier switching of the anti-CSP response to all measured IgG subclasses—IgG1 (Fig 3a), IgG2b (Fig 3b), IgG2c (Fig 3c), and IgG3 (Fig 3d) and again reflected systemic, not local, pre-existing immunity (S3 and S4 Figs).

Pre-vaccination with OVA_323-339_ stimulated production of the T_H_1 cytokine IFNγ (S2a Fig)) and the anti-OVA_323-339_ antibody response was skewed towards production of the T_H_1 isotype IgG2c [14] [15] [16] (686.5 ng/mL) over the T_H_2 isotype IgG1 (21.5 ng/mL, Fig 3e). The production of anti-CSP IgG2c was elicited particularly rapidly in OVA_323-339_ pre-vaccinated mice (Fig 3c) and reached higher levels (339.8 ng/mL vs 99.8 ng/mL), whereas IgG1 dominated the anti-CSP response in PBS pre-vaccinated animals (88.5 ng/mL vs 24.0 ng/mL, Fig 3e). This supports the possibility that the T_H_1/T_H_2 polarisation of the non-cognate CD4+ T cell population can influence the isotype of antibody elicited using this vaccine strategy.

CD4+ T cell help is also important for B cell somatic hypermutation and affinity maturation [3] [4]. We therefore assessed whether anti-CSP antibody responses to CSP(OVA_323-339_) vaccination would be of higher avidity when supported by pre-existing anti-OVA_323-339_ immunity. To measure antibody avidity, the chaotropic agent sodium thiocyanate (NaSCN) was used to disrupt weak antibody-antigen interactions [17]. Serum from mice pre-vaccinated with OVA_323-339_ contained higher avidity anti-CSP antibodies, measured by the concentration of NaSCN required to reduce the anti-CSP ELISA signal by 50%, compared with serum from mice that were not pre-vaccinated (1.29 M, vs 0.91 M, Fig 3f).

These data support the hypothesis that pre-existing anti-OVA_323-339_ CD4+ T cell responses can influence the isotype profile and increase the avidity of anti-CSP antibody responses following vaccination with CSP(OVA_323-339_).

### Encapsulation of Toll-like receptor 9 (TLR9) agonists to give self-adjuvanting particles can further enhance immunogenicity

For an antigen-driven B cell response to fully mature, two sequential antigen-dependent signals are required. In this model, BCR crosslinking by its target antigen is designated ‘signal one’, and results in efficient antigen uptake, processing, and presentation on MHCII [3]. MHCII-epitope complexes can then interact with CD4+ T cells at an immunological synapse, which results in the delivery of ‘signal two’ via co-stimulatory molecules and soluble mediators such as cytokines [18]. The CSP(OVA_323-339_) particles described above were designed to deliver signals one and two. However, entrapment of TLR ligands within the liposome could act as an important third signal enhancing naïve B cell responses to antigen [4] [19].

TLR9 is widely expressed in different murine B cell subsets and TLR9 agonism can stimulate proliferation and immunoglobulin secretion [20] [21]. TLR9 agonists have emerged as important adjuvants that stimulate a T_H_1-biased immune response [22] [23]. To further improve vaccine responses, DNA oligonucleotides with unmethylated CpG motifs were incorporated alongside OVA_323-339_ in the core of liposomal particles and designated CSP(OVA_323-339_+CpG) to generate ‘self-adjuvanting’ vaccine particles. CpG DNA could be demonstrated in liposomal vaccine preparations with a lipid-permeable oligonucleotide-binding fluorescent dye (S4a Fig). CpG DNA—either as free oligonucleotides (S4b Fig) or incorporated in CSP(OVA_323-339_+CpG) liposomes (S4c Fig)–was capable of activating murine TLR9 in a reporter cell assay.

**Fig 4:**
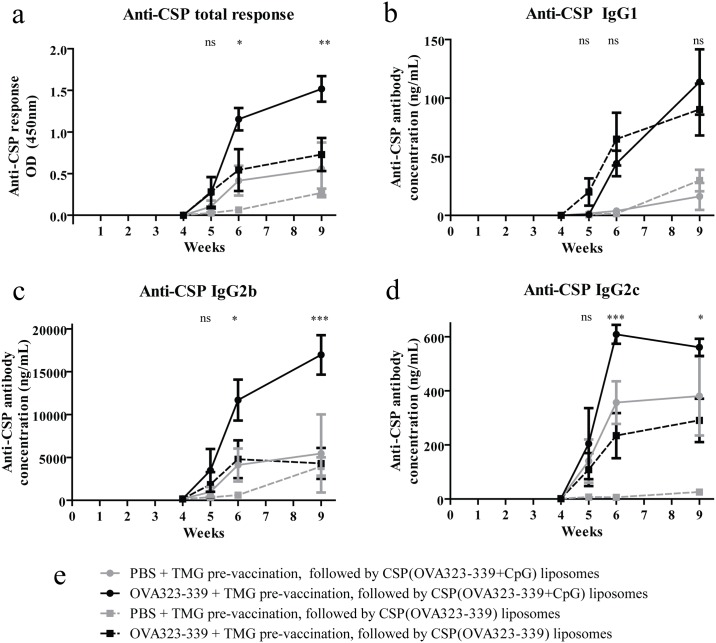
Particle-encapsulated TLR9 agonists further enhance immunogenicity. Liposomal particles were produced to contain CpG DNA TLR9 agonists in addition to OVA_323-339_ peptides. Mice (n = 4) were vaccinated with either PBS or OVA_323-339_ in TMG and then administered CSP(OVA_323-339_) or CSP(OVA_323-339_+CpG) liposomes. The total anti-CSP antibody response was compared between groups (a). The isotype of the anti-CSP response was quantified for IgG1 (b), IgG2b (c), and IgG2c (d). Mean responses in groups pre-vaccinated with OVA_323-339_ and then vaccinated with liposomes with or without CpG were compared (solid and dashed black lines) using two-way ANOVA with Bonferroni’s post test. Asterisks indicate the difference between groups shown in blue and grey groups at the indicated time points.

The presence of CpG DNA entrapped within the liposomal vaccine further augmented the effect of pre-existing anti-OVA_323-339_ CD4+ T cell immunity on anti-CSP antibody responses (Fig 4a). The inclusion of CpG DNA did not significantly alter the production of the T_H_2 isotype IgG1, which was primarily influenced by the presence or absence of pre-existing anti- OVA_323-339_ CD4+ T cells (Fig 4b). However, CSP(OVA_323-339_+CpG) liposomes elicited more IgG2b (16967 ng/mL vs 4303 ng/mL) and the T_H_1 isotype IgG2c (560.6 g/mL vs 290.6 ng/mL) when compared with CSP(OVA_323-339_) vaccination at week nine (Fig 4c and 4d).

These data indicate that incorporating CpG DNA in liposomal vaccine particles can further augment the enhanced—and T_H_1-biased—antibody response achieved by exploiting pre-existing vaccine-induced non-cognate CD4+ T cell immunity.

### Pre-existing anti-viral CD4+ T cells can be repurposed to support vaccine responses

The majority of the human population are chronically infected with HCMV, though rates vary with geography and detection methodology. Seroprevalence increases with age from 36% in 6–11-year-olds to 91% in those over 80 years old [9]. In chronic HCMV infection, a significant proportion of the CD8+ and CD4+ T cell pools recognise viral antigen. Approximately one third of infected adults have HCMV-specific populations comprising >20% of their circulating CD4+ and/or CD8+ memory T cell repertoire. Approximately 5% of the CD4+ T cell pool is directed against HCMV epitopes in healthy seropositive individuals, but this can be as high as 40% [10] [11] [12]. Individual clones can represent 0.3–1.5% of the total peripheral CD4+ T cell population [13]. These cells have a memory phenotype and secrete the T_H_1 cytokines IFNγ and TNFα [12] [24]. This significant and potentially powerful population of anti-CMV helper CD4+ T cells has not previously been exploited in humoral vaccine strategies.

Murine cytomegalovirus (MCMV) is a widely used murine model for HCMV and infection with MCMV leads to lifelong latency with sporadic, low level replication [25] [26]. The CD4+ T cell response to the MCMV m09_133-147_ epitope (here referred to as m09) was described as inflationary with 0.11–0.21% of the peripheral CD4+ T cell population producing IFNγ in response to m09 stimulation in persistently infected animals (days 40–100 post-infection) [27]. Here, we produced CSP-coated vaccine particles that encapsulated this MCMV m09 epitope—designated CSP(m09) liposomes—to assess whether anti-CMV CD4+ T cells could support anti-CSP vaccine responses.

**Fig 5:**
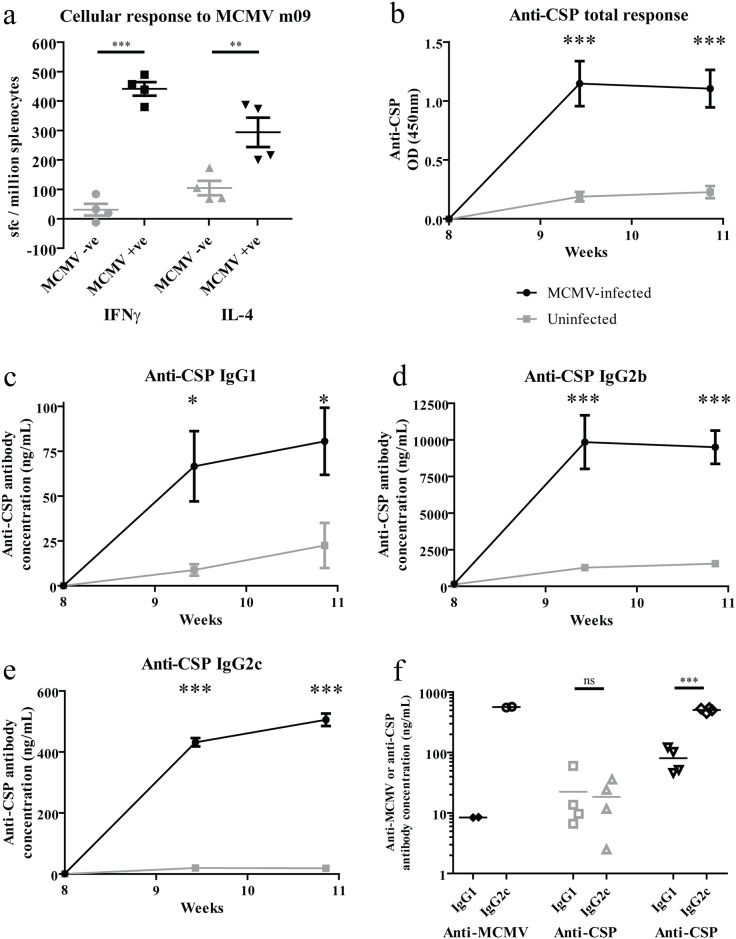
Non-cognate anti-viral CD4+ T cell populations in mice chronically infected with murine cytomegalovirus can enhance antibody production against target antigens. Mice (n = 4) were infected with MCMV. IFNγ and IL-4 responses to the MCMV m09 peptide were measured by ELISPOT assay in splenocytes from mice infected with MCMV for 8 weeks, or non-MCMV-infected matched controls and compared using unpaired, two-tailed t tests (a). 8 weeks post-infection, mice—including uninfected matched controls—were vaccinated with CSP-coated liposomes containing the m09 peptide. Each vaccine dose contained 0.5 μg of CSP and 0.1 μg of m09 peptide. The total anti-CSP response and CSP-specific IgG1 (c), IgG2b (d), and IgG2c (e) were quantified. Responses were compared using two-way ANOVA with Bonferroni’s post-test. The IgG1/IgG2c profile of the anti-MCMV response was also assessed by ELISA (n = 2) and is presented alongside anti-CSP IgG1 and anti-CSP IgG2c levels from vaccinated infected and uninfected mice (n = 4) with levels compared using unpaired, two-tailed t tests (f).

Splenocytes from mice infected with MCMV secreted both IFNγ and IL-4 in response to m09_133-147_ stimulation (Fig 5a). Following vaccination with CSP(m09) liposomes, anti-CSP responses were higher in chronically MCMV-infected mice when compared to uninfected controls (Fig 5b). MCMV infection can lead to non-specific hyper-responsiveness to unrelated antigens [28]. However, enhanced responses in MCMV infected mice were specific to m09-containing liposomes (S6 Fig). Anti-CSP IgG1 (80.5 ng/mL vs 22.5 ng/mL, Fig 5c), IgG2b (9506 ng/mL vs 1550 ng/mL, Fig 5d), and IgG2c (505.7 ng/mL vs 18.5 ng/mL, Fig 5e) levels were higher in MCMV-infected mice 20 days post-vaccination, indicating that this vaccine strategy elicited higher anti-CSP antibody responses in the context of chronic MCMV infection. To better appreciate the T_H_1/T_H_2 balance of the anti-MCMV and anti-CSP responses, the IgG2c/IgG1 profile of the response to MCMV prior to vaccination and the CSP response following vaccination were measured in infected and uninfected mice (Fig 5f). IgG2c production dominated the anti-MCMV antibody response (IgG1:IgG2c ratio 0.015) and also dominated the anti-CSP response following CSP(m09) vaccination in MCMV-infected mice (IgG1:IgG2c ratio 0.159), whereas the IgG2c/IgG1 response to CSP(m09) vaccination was more balanced in non-MCMV infected mice (IgG1:IgG2c ratio 1.215). As with the repurposing of anti-OVA T cells, (Fig 4), the effect of MCMV CD4+ T cells on anti-CSP antibody responses could be beneficially influenced by using liposomal vaccine particles that also encapsulated CpG DNA (S7 Fig).

These data indicate that anti-MCMV CD4+ T cells raised during chronic infection can be co-opted to enhance anti-CSP antibody production following the administration of CSP(m09) liposomes. This validates our primary hypothesis and provides an potential strategy for increasing the speed and potency of humoral vaccination in humans without the need for a pre-treatment step.

## Discussion

In this study, we describe a novel liposomal vaccine particle platform that spatially separates target and helper antigens, thereby binding to target-specific B cells and engaging help from pre-existing CD4+ T cells that recognise the encapsulated helper antigens. Liposomal vaccine particles were engineered to display the target B cell antigen CSP on their surface and to encapsulate CD4+ T cell helper epitopes from either Ovalbumin or MCMV, representing CD4+ cells induced by prior vaccination or chronic viral infection, respectively. These vaccine particles were able to repurpose pre-existing CD4+ T cell populations that recognised the core epitopes to support anti-CSP antibody responses. Engaging pre-existing non-cognate CD4+ T cell help stimulated the production of higher-titre, higher avidity, isotype-switched antibodies against the CSP antigen displayed on the particle surface. While pre-existing immune responses have been exploited by conjugate vaccines in the past, responses against carrier antigens can dominate. By encapsulating our helper CD4+ T cell epitopes within the vaccine particle core, antibody responses could be focused exclusively on the target antigen, overcoming the issue of helper antigen dominance. A similar concept can operate in influenza infection, with pre-existing CD4+ T cell responses to core viral epitopes shown to enhance antibody responses to envelope glycoproteins [29] [30]. However, this principle has not previously been translated into mechanism-driven particle vaccine design. A review of clinical studies with tetravalent Dengue vaccine particles (TDV) supports the hypothesis that pre-existing immunity to core vaccine components can enhance antibody responses to surface glycoproteins. The most advanced Dengue vaccine is a TDV based on a vector from the related flavivirus yellow fever virus (YFV) pseudotyped with glycoproteins from each of the four Dengue virus (DENV) serotypes. High levels of immunity to the YFV vaccine, or immunity YFV itself, correlates with enhanced antibody responses to DENV glycoproteins in small immunogenicity studies [31] and larger field trials [32] [33] [34]. While these vaccine particles were not designed to exploit this mechanism of action, it is encouraging to see real-world clinical data in accordance with our hypothesis and mouse model data. The critical advantages of the system described here are that one easily synthesised platform HCMV encapsulating vector could be easily and rapidly surface modified with any antigen of choice. Such a system provides a level of adaptability and ease of manufacture not associated with viral based vaccines.

In this study, we also demonstrate that the T_H_1/T_H_2 polarisation of the pre-existing CD4+ T cell population influenced the isotype of antibody produced against the non-cognate target antigen (Figs 3e and 5f). This suggests that the isotype profile of an antibody response to target antigens could be tuned by selecting which pre-existing non-cognate CD4+ T cell population to engage. An additional approach to control the magnitude and isotype of the antibody response was demonstrated by the inclusion of TLR9 agonists in liposomal vaccine particles. Particles could be engineered to include multiple different TLR agonists to tune the resulting immune response to combat particular pathogens.

Engaging non-cognate CD4+ T cell help led to rapid anti-CSP IgG antibody production that reached peak levels as early as one week after vaccination. While the response kinetics vary for different vaccines, IgM responses typically peak around one week post-vaccination whereas IgG responses take two to three weeks to reach their peak [35]. The ability of this platform to elicit rapid antibody production indicates it has utility in eliciting responses to target antigens in situations where swift responses are needed, such as vaccination in post-exposure prophylaxis or in a developing pandemic. This approach could also be useful in cancer vaccinology, which often faces additional hurdles of targeting ‘modified self’ antigens where a humoral response is likely to be limited by CD4+ T cell deletion or tolerance.

This paper is a proof-of-concept study using liposomal vaccine particles that were designed to harness helper signals from pre-existing non-cognate CD4+ T cells. Our findings suggest it should be possible to repurpose different CD4+ T cell populations in this way, to exploit pre-existing natural or vaccine-induced immunity extant in target populations. We identify three areas of importance for future work. Firstly, additional mechanistic studies are required to characterise the population of T helper cells that augment B cell responses to our vaccine particles. Secondly, studies looking at the duration of antibody response and the efficacy of this vaccine approach in a mouse model of malaria infection (such as a challenge study with *Plasmodium berghei* parasites expressing the NANP repeat of *Plasmodium falciparum*). Thirdly, more translationally relevant studies are required with helper epitopes that are relevant in human populations. We are now generating vaccine particles that encapsulate helper epitopes of potential utility in translational studies, such as Tetanus toxoid, Diptheria toxoid, Measles Virus, or HCMV epitopes, or combinations of epitopes (to account for the diversity of human MHCII molecules) [36]. Successful repurposing of pre-existing anti-CMV immunity is particularly appealing and may be useful in geographic areas where CMV seroprevalence is high, even in young children [37], or in elderly populations where CMV-specific CD4+ T cells reach very high levels [10][11][12]. For this reason, future studies could specifically look to target cancer antigens or important pathogens in elderly patients.

The rapid response and potential to elicit humoral responses that are otherwise limited by a deficiency in cognate CD4+ T cells mean this approach could have important implications for future rationally designed humoral vaccine strategies.

## Materials and Methods

### Animal experiments

Female C57Bl/6 mice aged 6–8 weeks at the start of experiments were housed in pathogen-free conditions at the University of Oxford animal facility. Mice were housed (6 animals per cage) with a 12/12 hour light/dark cycle, with ad libitum access to food and water, appropriate bedding, and with regular waste disposal. When indicated, mice were anaesthetised with isoflurane and then vaccination was performed either subcutaneously or intramuscularly. At the appropriate time points, blood was obtained by sampling 20 μL from the tail vein. Upon completion of a given study, mice were euthanized by cervical dislocation under terminal isoflurane anaesthesia. These experiments were conducted in a dedicated facility with daily monitoring (appearance, activity level, etc) from qualified animal research technicians. Animal weights were measured at least weekly. All animal experiments were performed under valid United Kingdom Home Office project (PPL 30/2819) and personal (PIL 40/10329) licenses and were conducted in accordance with the terms of the United Kingdom Home Office guidelines and with the approval of the Medical Sciences Animal Ethics Committee, University of Oxford.

### Liposome formulation

Liposomal particles were produced using the lipid film hydration method originally described by Bangham [38]. Briefly, lipids as powder were individually dissolved in a (2:1 chloroform:methanol) solvent mix and then mixed in appropriate quantities in a round bottom flask, to achieve desired molar ratios and total final lipid concentrations of 20–40 mg/mL. Liposomes were prepared to contain 43 mol% DMPC (1,2-dimyristoyl-sn-glycero-3-phosphocholine, Avanti Polar Lipids, USA), 42 mol% Cholesterol (Sigma Aldrich, UK), 10 mol% DSPE-PEG2000-maleimide (1,2-distearoyl-sn-glycero-3-phosphoethanolamine-N-[maleimide(polyethylene glycol)-2000], Avanti Polar Lipids, USA), and 5 mol% DMPG (Avanti Polar Lipids, USA).

A LABOROTOR 4011 rotary evaporator and water bath (Heidolph Instruments, Germany)–set to 60 rpm, 45°C, and 200 mmHg vacuum—was used to remove the solvent over a period of up to 30 minutes. Trace solvent was then removed from the resulting thin lipid film using a high vacuum pump for at least one hour. This film was then hydrated with a 1 mL of citrate (20 mM, pH 6.3, [used for OVA_323-339_ studies]) or HEPES (20 mM, pH 8.2, [used for m09 studies]) buffer containing the relevant peptide or CpG DNA (both at 1 mg/mL) using the rotary evaporator at 80 rpm and 45°C without vacuum, under argon. The resulting multi-lamellar lipid and antigen/adjuvant preparation was then extruded at 50°C with eleven passes through a 400 nm polycarbonate membrane (Avanti Polar Lipids, USA) using 1 mL glass syringes (Hamilton Company, USA) and a mini-extruder (Avanti Polar Lipids, USA). This process was repeated with eleven passes through a 200 nm polycarbonate membrane (Avanti Polar Lipids, USA) before the resulting liposomes were purified using a PBS-washed Sephadex G-25 PD10 size exclusion column (GE Healthcare, UK). Liposomal preparations, now in PBS solution, were then immediately added to 10 mg/mL CSP antigen and left to react overnight at room temperature prior to dialysis. Dialysis was performed using 20,000 MWCO Slide-A-Lyzer cassettes (Pierce, USA) in 1 L of PBS at 4°C with gentle stirring of the dialysis buffer. After two hours, the initial buffer was replaced and the dialysis proceeded overnight. The following day, the buffer was again changed and dialysis continued for another hour before the sample was isolated by piercing the cassette with a needle and syringe.

### Antigens

Antigens were synthesised by custom peptide synthesis (ThermoScientific, Germany).

### Dynamic light scattering

A Zetasizer Nano ZS instrument (Malvern, UK) was used to analyse samples diluted 1:50 in PBS up to a 1 mL volume in UV grade cuvettes (Fisher Scientific, UK). Data was analysed using Malvern Zetasizer software v6.32.

### Flow cytometric analysis of particles

A FACSCalibur flow cytometer (Becton Dickinson, San Jose, Calif.) equipped with a 15-mW 488-nm argon-ion laser was used to characterise viral particles and liposomes in solution. Measurement of PBS buffer alone defined the size region where noise was observed and it was established that liposomal particles gave a FSC/SSC particle distinct from their suspension buffers and that these populations could be gated to allow analysis of fluorescence. Particle-associated OVA_323-339_ was assessed by producing liposomes with FITC-labelled OVA_323-339_ peptides and measuring particle fluorescence by flow cytometry. Particle-associated CSP was detected by staining particles with anti-CSP antibodies (mouse mAb 2A10 –a kind gift of Dr Simon Draper, Jenner Institute, University of Oxford) followed by staining with a FITC-labelled goat anti-mouse secondary antibody (Sigma-Aldrich, UK, F2772). Antibody staining steps were performed with agitation at room temperature for one hour. Flow cytometric analysis was performed on a FACSCalibur flow cytometer (Becton Dickinson, USA) and analysed using FlowJo software (Tree Star, USA).

### Vaccination of mice to establish pre-existing CD4+ T cell populations

Mice were vaccinated with 10 μg of OVA_323-339_ peptide, or PBS, emulsified 1:1 in a total volume of 100 μL of TiterMax^®^ Gold (TMG) by vortexing. Vaccination was performed subcutaneously, except where the intramuscular route is indicated. For intramuscular injections, 10 μg of OVA_323-339_ or PBS was emulsified 1:1 in a total volume of 20 μL of TMG.

### ELISPOT assays

Spleens were harvested from sacrificed mice and manually crushed before filtration through a 70 μm cell strainer (Becton Dickinson, USA). 10 mL of RPMI media with 10% FCS was added and cells were spun at 1350 rpm for 5 minutes in an Eppendorf 5810R centrifuge with an Eppendorf A-4-62 rotor. Red blood cells were lysed by re-suspending pelleted cells in 5mL blood cell lysis buffer (Sigma-Aldrich, UK) for 5 minutes at room temperature. Lysis was stopped with the addition of 25 mL PBS before cell suspensions were spun again at 1350 rpm for 5 minutes. The resulting cell pellet was re-suspended in 10 mL media for cell counting and use in subsequent assays.

High protein binding PVDF 96 well plates (Millipore, UK) were briefly activated with 50 μL of 70% ethanol per well, then washed five times with sterile PBS. Plates were coated with 50 μl diluted capture antibody (15 μg/mL in PBS) and incubated overnight at 4°C. Excess capture antibody was removed by washing five times with sterile PBS which was replaced with RPMI containing 10% FCS for one hour at 37°C to prevent non-specific binding. Blocking media was removed and replaced with isolated splenocytes at 1x10^5^–3.3x10^5^ cells per well, depending on the experiment. Antigenic stimuli were applied to cells in triplicate at a final concentration of 1μg/mL. Concanavalin A Type VI (Sigma-Aldrich, UK) at 1 μg/mL was used as a positive control.

Plates were incubated at 37°C for 24–48 hours then washed five times with PBS. 50 μl of biotinylated detection antibody (1 μg/mL with 0.5% FCS and 0.2μm filtered to reduce background) was added to each well. Plates were covered in foil and left for two hours at room temperature before washing five times with PBS. 50 μL streptavidin-ALP (1 μg/mL in PBS with 0/5% FCS) was added per well and incubated at room temperature for 45 minutes. After washing plates five times with PBS, 50 μL of BCIP/NBT colour development buffer (Sigma-Aldrich) was added to each well. Plates were left at room temperature until spots developed (approximately 20–30 minutes) and then washed with water to stop the reaction. Plates were allowed to dry and then read in an ELISPOT plate reader. Spots were counted automatically using AID software (Autoimmun Diagnostika, Germany). For analysis, the number of background spots (PBS treated) for each sample was subtracted from the antigen-treated well values and the total number of spots was presented as per million splenocytes.

### Vaccination of mice with liposomal vaccines

Vaccination was performed with 100 μL volumes, via the subcutaneous route, unless otherwise specified. Each vaccine dose contained 0.5 μg of CSP and 0.1 μg of OVA_323-339_, with or without 0.1 μg of CpG DNA (Fig 4).

### Serum antibody measurements

High protein binding MaxiSorp 96 well plates (Nunc, Denmark) were coated with 50 ng if antigen in 50 μL PBS per well and incubated at 4°C overnight. Unbound antigen was removed by washing six times with PBS/0.05% Tween (Fisher Scientific, UK). Plates were then blocked with 5% Bovine Serum Albumin (BSA) (Sigma-Aldrich, UK) diluted in PBS for one hour at room temperature. Blocking solutions were removed and replaced with serum samples from vaccinated mice and, where necessary, primary antibodies as positive controls. Serum samples were diluted to 1:1000, unless otherwise indicated. Plates were then incubated at room temperature for two hours. Serum was removed and plates were washed six times with PBS/0.05% Tween. Secondary antibody (anti-mouse Ig HRP) was added to the plate (50 μL/well at a 1:5000 dilution) and incubated for one hour at room temperature. Secondary antibody was removed and the plate was again washed six times with PBS/0.05% Tween. HRP substrate 1-Step UltraTMB (Fisher Scientific, UK) was added to the wells (50 μL/well) and the reaction was stopped after an appropriate time with 50 μL/well acid stop solution (Alpha Diagnostics). Absorbance was measured at 450 nm using a Wallac 1420 Victor2 plate reader (PerkinElmer). For data analysis, background values (determined from control wells e.g. those incubated with PBS in place of serum) were subtracted from raw data, to give net OD values.

### Antigen-specific isotype measurement

To measure the isotype of antigen-specific antibody responses, plates were coated with antigen in PBS, washed, blocked with BSA, incubated with serum, and washed as described above. Biotinylated anti-isotype secondary antibodies (Biolegend, UK) were then added (50 μL per well at 1:1000 dilution) to detect the presence of specific isotypes. Plates were then washed six times with PBS/0.05% Tween and streptavidin-HRP (Invitrogen, UK) was then added (50 μL per well at 1:5000 dilution). Plates were washed six times with PBS/0.05% Tween prior to development with HRP substrate as described above. Plates coated in serial dilutions of purified isotypes (Biolegend, UK) were also prepared and developed as described here to give standard curves that allowed the quantification of antigen-specific isotype responses.

### Sodium thiocyanate antibody avidity assay

Antibody avidity assays were performed using the chaotropic agent sodium thiocyanate (NaSCN) to disrupt antibody-antigen interactions [17]. This assay was performed in an ELISA plate format using increasing concentrations of NaSCN (diluted in PBS). After CSP-coated ELISA plates were washed, blocked with BSA, incubated with serum from vaccinated mice (individually diluted to a level calculated to give an OD450 of 1.0), and washed five times with PBS/0.05% Tween, NaSCN-treated wells were exposed to ascending concentrations (0-5M) of NaSCN at room temperature for 10 minutes. Negative control wells were treated with PBS only. Plates were then washed five times with PBS/0.05% Tween and the presence of CSP-specific mouse antibodies was detected as described in section 2.12.1. Resistance to thiocyanate elution was used as an indicator of avidity and the concentration of NaSCN required to reduce the ELISA signal by 50% was used to compare different vaccination strategies.

### TLR9 agonists

The oligonucleotide sequence 5’-tccatgacgttcctgacgtt-3’ was synthesised (Sigma, UK) with a phosphodiester backbone. This was incorporated in liposomes alongside peptide epitopes, as described previously.

### HEK-Blue-mTLR9 reporter cells

Reporter cells were obtained from Invivogen and used as per the manufacturer’s instructions. This cell was produced by co-transfection of the murine TLR9 gene and an inducible, secreted form of embryonic alkaline phosphatase (SEAP). SEAP expression is under the control of the IFN-β minimal promoter fused to five AP-1 and NF-κB binding sites. HEK-Blue^™^ media (Invivogen, France) was used to quantify SEAP expression by spectrophotometry (OD 625nm).

### MCMV-infection

The Smith strain of murine cytomegalovirus (MCMV) was used as a model of chronic cytomegalovirus in mice. To establish infection, 1x10^5^ pfu of MCMV was injected intravenously into mice 8 weeks prior to vaccination with liposomal particles.

### Anti-MCMV ELISA

Antibody responses to MCMV were measured using the MCMV antigen-coated wells of commercially available ELISA plates (Xpress Bio, USA). ELISAs were performed as previously described.

### Statistics and graphs

All data are represented as mean ± standard error of the mean (SEM). Statistical analyses were performed using Prism software (GraphPad). Means were compared with unpaired, two-tailed t tests, or one/two-way analysis of variance (ANOVA) and Tukey or Bonferroni post tests were used, as indicated. Differences were found to be significant when p value was less than 0.05. Indicators of statistical significance: ns, not significant (p > 0.05); *, p < 0.05; **, p < 0.01; ***, p < 0.001).

## Supporting Information

S1 Fig ELISPOT characterisation of the anti-OVA CD4+ T cell response in mice vaccinated with OVA_323-339_ peptides in TMG. 6–8 week old female C57Bl/6 mice (n = 3) were administered two subcutaneous vaccinations of 10 μg of OVA_323-339_ peptide or PBS emulsified in TiterMax^®^ Gold adjuvant, with a two week interval between doses. Two weeks after the second vaccination, splenocytes were harvested and stimulated with OVA_323-339_ peptide at a final concentration of 1 μg/mL. 36 hours later, cellular responses to IFNγ (A and B) and IL-4 (C and D) were measured by ELISPOT assay and the number of spot forming cells (sfc) was counted for each well (n = 3 wells per mouse). Technical triplicates were averaged and mean responses were calculated for each group before comparison with unpaired, two-tailed t tests.
(TIF)

S2 Fig Liposomes can be produced to mimic viral particles with surface-bound target antigens and encapsulated CD4+ T cell epitopes. Liposomal particles were generated to contain OVA_323-339_ epitopes in the particle core and the B cell antigen of *Plasmodium falciparum* on the particle surface—designated CSP(OVA_323-339_) liposomes. (A) The size and polydispersity of CSP(OVA_323-339_) liposomes was assessed by dynamic light scattering. (B) Encapsulation of OVA_323-339_ was confirmed by evaluation of particles produced with FITC-labelled OVA_323-339_ in a flow cytometer. (C) Surface-bound CSP was detected with anti-CSP monoclonal antibody and flow cytometric analysis of liposomal particles. DLS and flow cytometry results are representative of multiple experiments and results of typical experiments are shown. (D) The functionality of liposomal vaccine particles was measured by ELISPOT. Splenocytes from mice (n = 3) that had been vaccinated twice with 10 μg of OVA_323-339_ in TiterMax^®^ Gold adjuvant were incubated with CSP(OVA_323-339_ liposomes. To generate antibody-coated liposomal particles, liposomal preparations were incubated for one hour at room temperature with 1:100 diluted ‘CSP-naïve serum’ (from mice vaccinated with OVA_323-339_ in TMG alone) or ‘CSP-immune serum’ (from mice also vaccinated with CSP-coated liposomes where anti-CSP antibodies were previously demonstrated by ELISA). IFNγ responses were measured by ELISPOT after 24 hours incubation and the influence of CSP-immune serum on CSP(OVA_323-339_) liposome particle-stimulated IFNγ production from splenocytes was assessed. Means (n = 3) were compared with unpaired, two-tailed t tests.
(TIF)

S3 Fig Effect of systemic immunity on subcutaneous vaccination. 6–8 week old female C57Bl/6 mice (n = 4) were administered two subcutaneous vaccinations of 10 μg of OVA_323-339_ peptide or PBS emulsified in TiterMax^®^ Gold adjuvant, or two intramuscular injections of 10 μg of OVA_323-339_ peptide in TiterMax^®^ Gold adjuvant, with a two week interval between doses Two weeks later, this was followed a single subcutaneous dose of CSP(OVA_323-339_) liposomes. The effect of pre-existing anti- OVA_323-339_ CD4+ T cell immunity, generated by subcutaneous or intramuscular vaccination, on the developing anti-CSP IgG1, IgG2b, and IgG2c antibody response was measured over four weeks.
(TIF)

S4 Fig Effect of systemic immunity on intramuscular vaccination. 6–8 week old female C57Bl/6 mice (n = 4) were administered two intramuscular vaccinations of 10μg of OVA_323-339_ peptide or PBS emulsified in TiterMax^®^ Gold adjuvant, or two subcutaneous injections of 10μg of OVA_323-339_ peptide in TiterMax^®^ Gold adjuvant, with a two week interval between doses. Two weeks later, this was followed a single intramuscular dose of CSP(OVA_323-339_) liposomes. The effect of pre-existing anti- OVA_323-339_ CD4+ T cell immunity, generated by subcutaneous or intramuscular vaccination, on the developing anti-CSP IgG1, IgG2b, and IgG2c antibody response was measured over four weeks.
(TIF)

S5 Fig Liposomal vaccine particles can be engineered to contain CpG DNA and these particles can stimulate TLR9. The presence of CpG DNA TLR9 agonists was measured in PD10 column fractions during purification of liposomes encapsulating CpG and the peptide OVA323-339. The presence of concentrated liposomes in fraction 4 was confirmed by DLS and these were reacted overnight with CSP antigen and then dialysed overnight before CpG content was measured by OliGreen assay (a). HEK-Blue-mTLR9 reporter cells were incubated for 24 hours with increasing concentrations of TLR9 agonist (b) or with CSP(OVA323-339 + CpG) liposomes, CSP(OVA323-339) liposomes, or CSP(empty) liposomes (c). SEAP expression levels were measured by detection of a colorimetric product from SEAP substrate-containing HEK-blue detection media.
(TIF)

S6 Fig Anti-CSP responses to lower dose vaccination with CSP(m09), CSP(scr m09), CSP9(m09+CpG), CSP(empty), and CSP(CpG) liposomes in uninfected and MCMV-infected mice. Female 6–8 week old C57Bl/6 mice were infected with MCMV or housed as uninfected controls. Eight weeks later, both groups were vaccinated subcutaneously with CSP(m09) liposomes containing 0.5 μg of CSP and, where indicated, 0.1 μg of m09, a scrambled peptide of the m09 amino acid sequence (‘scr m09’), and/or CpG DNA, in 100 μL volumes. Serum was collected at before liposomal vaccination and at days 10 and 20 after it. The effect of MCMV-infection on the production of anti-CSP immunoglobulin was measured by ELISA for each vaccine formulation (A-E). For each formulation, mean OD levels (+/- SEM) are displayed. Means were compared between MCMV-infected and uninfected groups using two-way ANOVA with Bonferroni’s post-test (n = 4).
(TIF)

S7 Fig Analysis of the isotype profile of anti-CSP antibodies elicited by vaccination with CSP(m09) or CSP(m0+CpG) liposomes in uninfected and MCMV-infected mice. Female 6–8 week old C57Bl/6 mice were infected with MCMV or housed as uninfected controls. Eight weeks later, both groups were vaccinated subcutaneously with CSP(m09) liposomes containing 0.5 μg of CSP and 0.1 μg m09 in 100 μL volumes. The effect of MCMV infection on the total anti-CSP IgG response (a) and the degree and speed of switching IgG1, IgG2b, and IgG2c was measured (b-d). Similarly, total anti-CSP IgG (e), IgG1 (f), IgG2b (g), and (IgG2c (h) were measured in MCMV-infected and uninfected mice vaccinated with CSP(m09+CpG) liposomes. Mean OD levels (+/- SEM) are displayed. Means were compared using two-way ANOVA with Bonferroni’s post-test (n = 4).
(TIF)
