# Supplementary figures and images for: A Rapid-Response Humoral Vaccine Platform Exploiting Pre-Existing Non-Cognate Populations of Anti-Vaccine or Anti-Viral CD4+ T Helper Cells to Confirm B Cell Activation
Source: PLoS One. 2016 Nov 18;11(11):e0166383. doi: 10.1371/journal.pone.0166383 (PMC5115735; doi:10.1371/journal.pone.0166383)

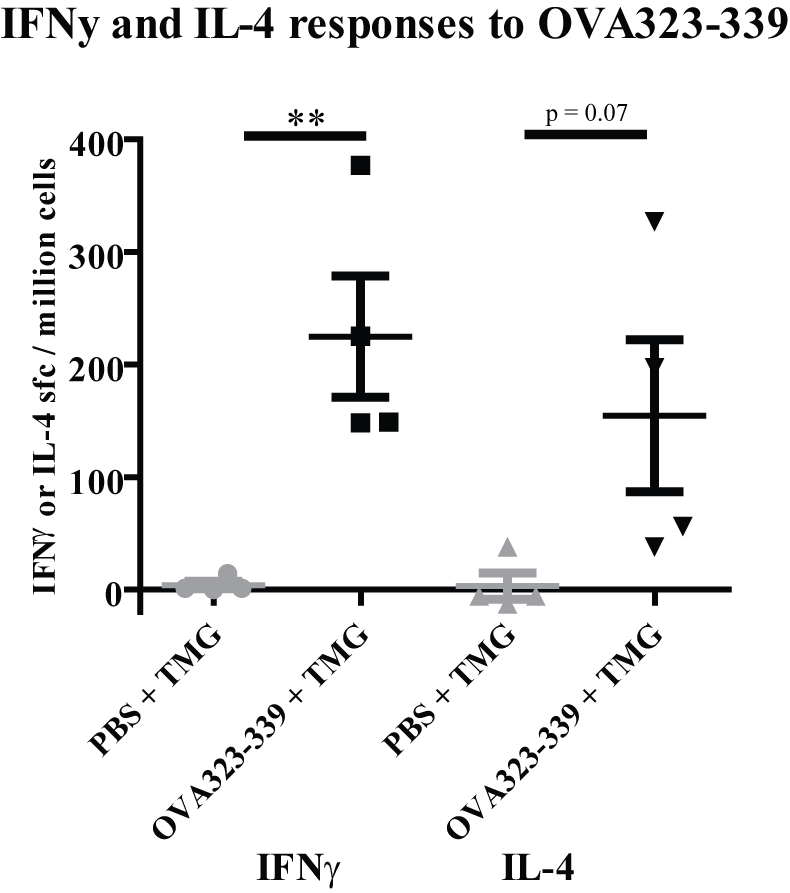

Supplement: S1 Fig — 6–8 week old female C57Bl/6 mice (n = 3) were administered two subcutaneous vaccinations of 10 μg of OVA323-339 peptide or PBS emulsified in TiterMax® Gold adjuvant, with a two week interval between doses. Two weeks after the second vaccination, splenocytes were harvested and stimulated with OVA323-339 peptide at a final concentration of 1 μg/mL. 36 hours later, cellular responses to IFNγ (A and B) and IL-4 (C and D) were measured by ELISPOT assay and the number of spot forming cells (sfc) was counted for each well (n = 3 wells per mouse). Technical triplicates were averaged and mean responses were calculated for each group before comparison with unpaired, two-tailed t tests. (TIF) [file pone.0166383.s001.tif]

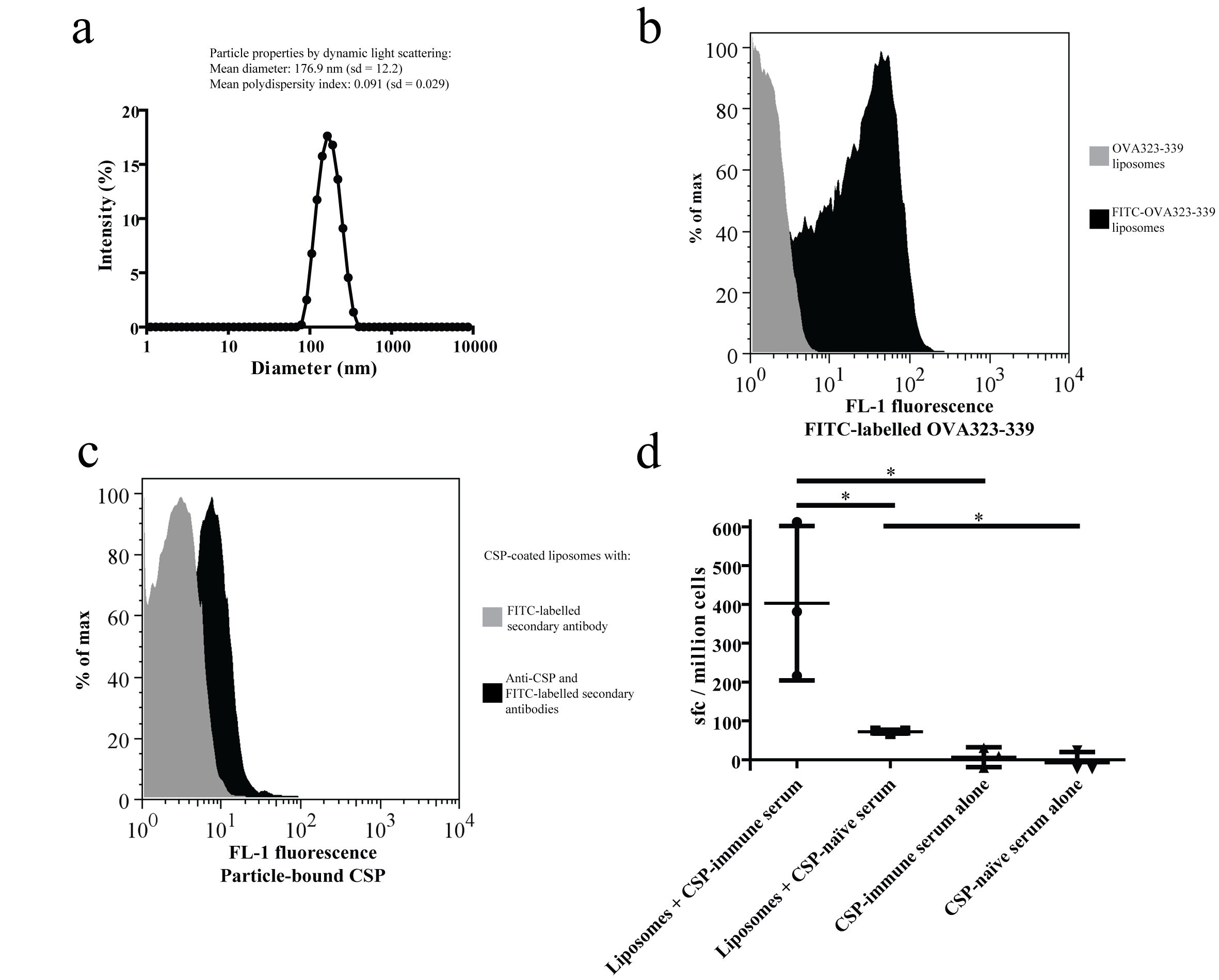

Supplement: S2 Fig — Liposomal particles were generated to contain OVA323-339 epitopes in the particle core and the B cell antigen of Plasmodium falciparum on the particle surface—designated CSP(OVA323-339) liposomes. (A) The size and polydispersity of CSP(OVA323-339) liposomes was assessed by dynamic light scattering. (B) Encapsulation of OVA323-339 was confirmed by evaluation of particles produced with FITC-labelled OVA323-339 in a flow cytometer. (C) Surface-bound CSP was detected with anti-CSP monoclonal antibody and flow cytometric analysis of liposomal particles. DLS and flow cytometry results are representative of multiple experiments and results of typical experiments are shown. (D) The functionality of liposomal vaccine particles was measured by ELISPOT. Splenocytes from mice (n = 3) that had been vaccinated twice with 10 μg of OVA323-339 in TiterMax® Gold adjuvant were incubated with CSP(OVA323-339 liposomes. To generate antibody-coated liposomal particles, liposomal preparations were incubated for one hour at room temperature with 1:100 diluted ‘CSP-naïve serum’ (from mice vaccinated with OVA323-339 in TMG alone) or ‘CSP-immune serum’ (from mice also vaccinated with CSP-coated liposomes where anti-CSP antibodies were previously demonstrated by ELISA). IFNγ responses were measured by ELISPOT after 24 hours incubation and the influence of CSP-immune serum on CSP(OVA323-339) liposome particle-stimulated IFNγ production from splenocytes was assessed. Means (n = 3) were compared with unpaired, two-tailed t tests. (TIF) [file pone.0166383.s002.tif]

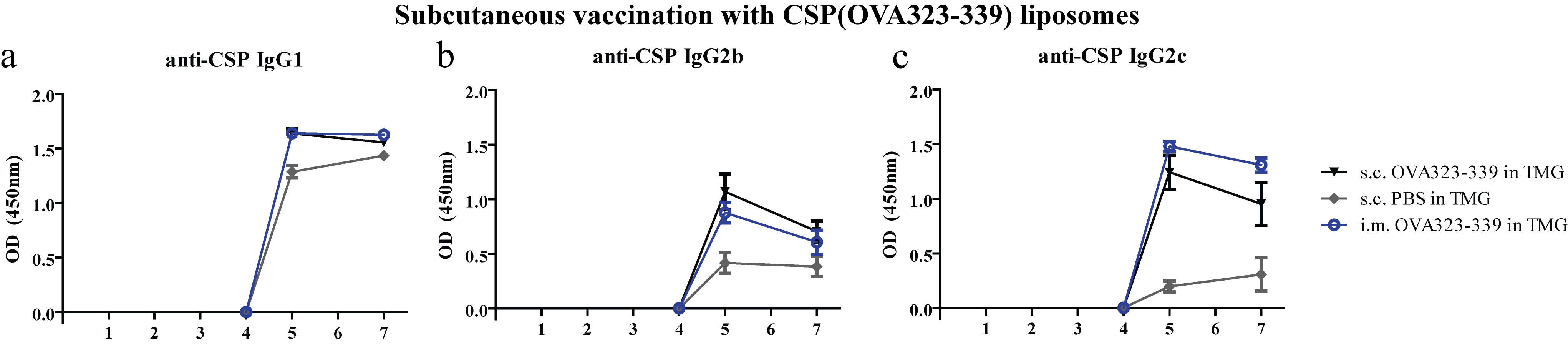

Supplement: S3 Fig — 6–8 week old female C57Bl/6 mice (n = 4) were administered two subcutaneous vaccinations of 10 μg of OVA323-339 peptide or PBS emulsified in TiterMax® Gold adjuvant, or two intramuscular injections of 10 μg of OVA323-339 peptide in TiterMax® Gold adjuvant, with a two week interval between doses Two weeks later, this was followed a single subcutaneous dose of CSP(OVA323-339) liposomes. The effect of pre-existing anti- OVA323-339 CD4+ T cell immunity, generated by subcutaneous or intramuscular vaccination, on the developing anti-CSP IgG1, IgG2b, and IgG2c antibody response was measured over four weeks. (TIF) [file pone.0166383.s003.tif]

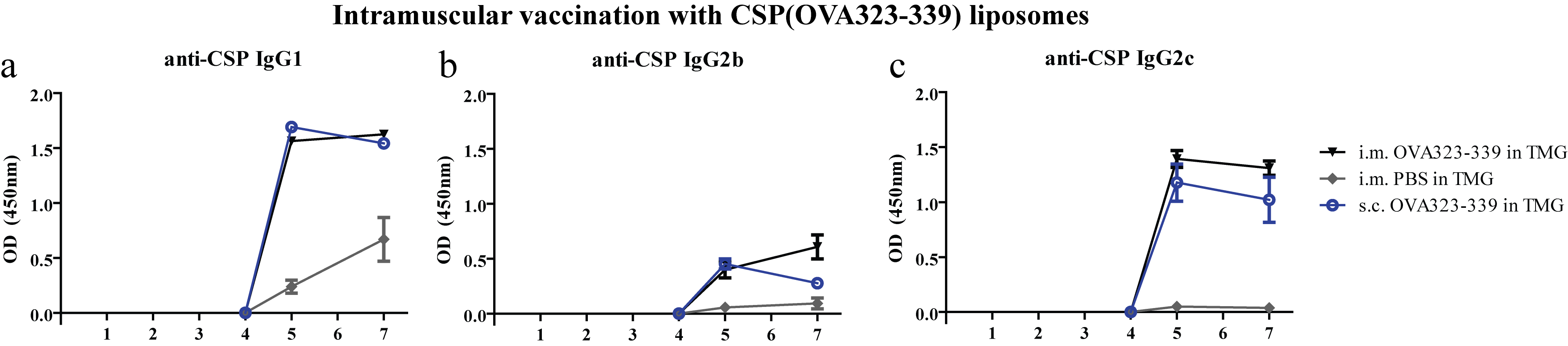

Supplement: S4 Fig — 6–8 week old female C57Bl/6 mice (n = 4) were administered two intramuscular vaccinations of 10μg of OVA323-339 peptide or PBS emulsified in TiterMax® Gold adjuvant, or two subcutaneous injections of 10μg of OVA323-339 peptide in TiterMax® Gold adjuvant, with a two week interval between doses. Two weeks later, this was followed a single intramuscular dose of CSP(OVA323-339) liposomes. The effect of pre-existing anti- OVA323-339 CD4+ T cell immunity, generated by subcutaneous or intramuscular vaccination, on the developing anti-CSP IgG1, IgG2b, and IgG2c antibody response was measured over four weeks. (TIF) [file pone.0166383.s004.tif]

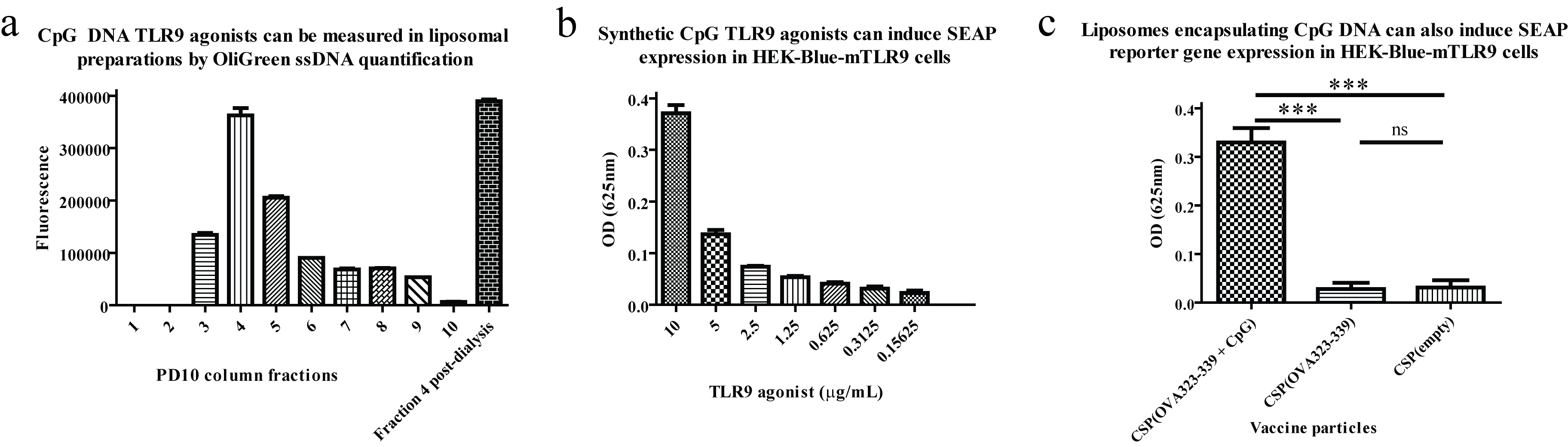

Supplement: S5 Fig — The presence of CpG DNA TLR9 agonists was measured in PD10 column fractions during purification of liposomes encapsulating CpG and the peptide OVA323-339. The presence of concentrated liposomes in fraction 4 was confirmed by DLS and these were reacted overnight with CSP antigen and then dialysed overnight before CpG content was measured by OliGreen assay (a). HEK-Blue-mTLR9 reporter cells were incubated for 24 hours with increasing concentrations of TLR9 agonist (b) or with CSP(OVA323-339 + CpG) liposomes, CSP(OVA323-339) liposomes, or CSP(empty) liposomes (c). SEAP expression levels were measured by detection of a colorimetric product from SEAP substrate-containing HEK-blue detection media. (TIF) [file pone.0166383.s005.tif]

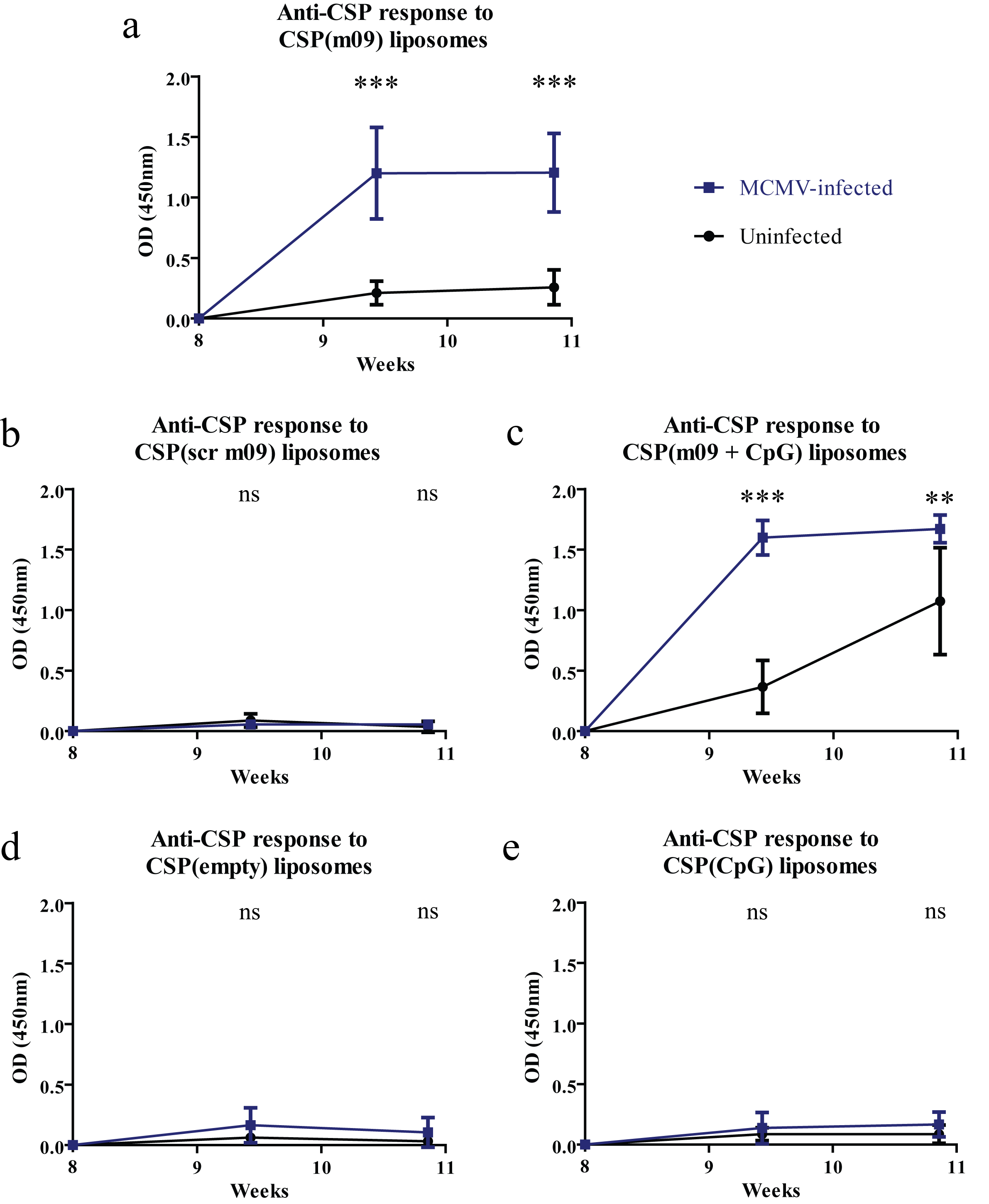

Supplement: S6 Fig — Female 6–8 week old C57Bl/6 mice were infected with MCMV or housed as uninfected controls. Eight weeks later, both groups were vaccinated subcutaneously with CSP(m09) liposomes containing 0.5 μg of CSP and, where indicated, 0.1 μg of m09, a scrambled peptide of the m09 amino acid sequence (‘scr m09’), and/or CpG DNA, in 100 μL volumes. Serum was collected at before liposomal vaccination and at days 10 and 20 after it. The effect of MCMV-infection on the production of anti-CSP immunoglobulin was measured by ELISA for each vaccine formulation (A-E). For each formulation, mean OD levels (+/- SEM) are displayed. Means were compared between MCMV-infected and uninfected groups using two-way ANOVA with Bonferroni’s post-test (n = 4). (TIF) [file pone.0166383.s006.tif]

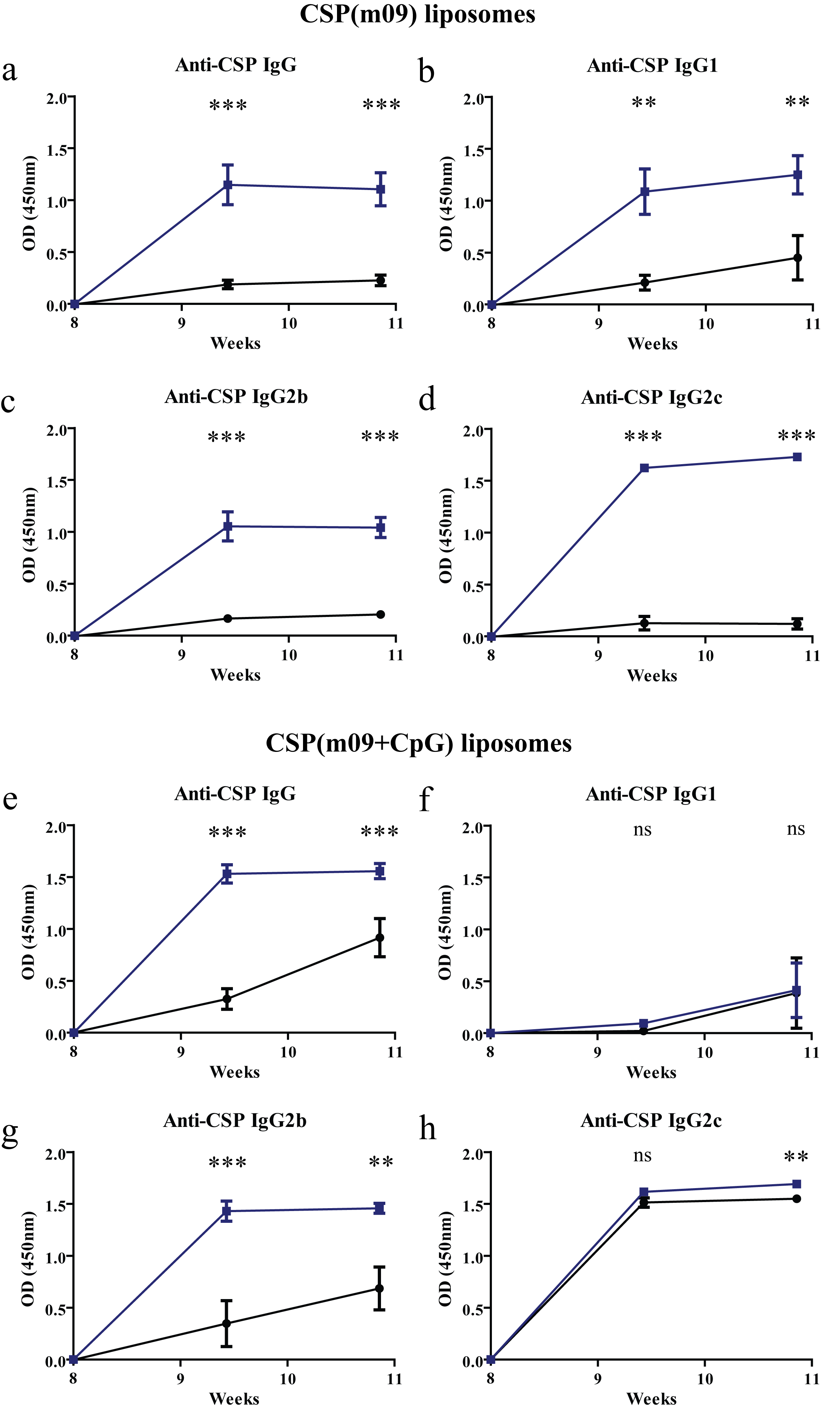

Supplement: S7 Fig — Female 6–8 week old C57Bl/6 mice were infected with MCMV or housed as uninfected controls. Eight weeks later, both groups were vaccinated subcutaneously with CSP(m09) liposomes containing 0.5 μg of CSP and 0.1 μg m09 in 100 μL volumes. The effect of MCMV infection on the total anti-CSP IgG response (a) and the degree and speed of switching IgG1, IgG2b, and IgG2c was measured (b-d). Similarly, total anti-CSP IgG (e), IgG1 (f), IgG2b (g), and (IgG2c (h) were measured in MCMV-infected and uninfected mice vaccinated with CSP(m09+CpG) liposomes. Mean OD levels (+/- SEM) are displayed. Means were compared using two-way ANOVA with Bonferroni’s post-test (n = 4). (TIF) [file pone.0166383.s007.tif]
